# Proteomics dataset containing proteins that obscure identification of TOPLESS interactors in *Arabidopsis*

**DOI:** 10.1016/j.dib.2018.08.059

**Published:** 2018-08-29

**Authors:** Joe Collins, Craig Dufresne, William B. Gurley, Sixue Chen

**Affiliations:** a Plant Molecular and Cellular Biology Program, University of Florida, Gainesville, FL, USA; b Department of Biology, Genetics Institute, Plant Molecular and Cellular Biology Program, University of Florida, Gainesville, FL, USA; c Thermo Fisher Scientific, West Palm Beach, FL, USA; d Department of Microbiology and Cell Science, University of Florida, Gainesville, FL, USA; e Proteomics and Mass Spectrometry, Interdisciplinary Center for Biotechnology Research, University of Florida, Gainesville, FL, USA

## Abstract

Here we report proteins identified after conducting Tandem Affinity Purification (TAP) of the TOPLESS (TPL) corepressor from *Arabidopsis*. We generated transgenic plants harboring TPL fused to the GS-TAG, “Boosting tandem affinity purification of plant protein complexes” (Van Leene et al., 2008) [1]. Four independent biological replicates of a selected TPL-GS-TAG line were grown simultaneously, crosslinked with formaldehyde, and proteins were isolated from whole plant tissue via TAP. Purified proteins were treated with trypsin, and the peptides were analyzed via mass spectrometry. Datasets are hosted in the MassIVE public repository (reference number: MSV000082477, https://massive.ucsd.edu/ProteoSAFe/dataset.jsp?task=f16255fb7080426a9fe1926b4d3d5862). The data in this article has not been published elsewhere and is original to this work.

**Specifications Table**

**Table:** 

Subject area	*Plant molecular biology*
More specific subject area	*Protein interaction network of the TOPLESS corepressor*
Type of data	*Table, image, figure*
How data was acquired	*Tandem affinity purification and LC–MS. EASY-nLC 1000 and Orbitrap Elite ETD (Thermo Fisher Scientific™)*
Data format	*Raw, filtered, analyzed*
Experimental factors	*Transgenic Arabidopsis expressing TOPLESS-GS-TAG*
Experimental features	*Tandem affinity purification was used to isolate TOPLESS to identify protein partners from 2 weeks old transgenic Arabidopsis.*
Data source location	*Gainesville, FL, United States*
Data accessibility	*Data is hosted in the MassIVE public repository (MSV000082477).*https://massive.ucsd.edu/ProteoSAFe/dataset.jsp?task=f16255fb7080426a9fe1926b4d3d5862
Related research articles	J. Van Leene, E. Witters, D. Inzé, G. De Jaeger. Boosting tandem affinity purification of plant protein complexes. Trends Plant Sci (2008) 13, 517–520 [1].
	J. Van Leene, D. Eeckhout, G. Persiau, E. Van De Slijke, J. Geerink, G. Van Isterdael, E. Witters, G. De Jaeger. Isolation of transcription factor complexes from Arabidopsis cell suspension cultures by tandem affinity purification. Methods Mol Biol (2011) 754, 195–218 [7].

**Value of the data**

• Many proteins associate into complexes that directly influence their activity in the cell, and identifying these protein interaction networks is an important resource for understanding protein function.
• The high sensitivity of mass spectrometry makes it a powerful tool to identify interacting proteins, but it also necessitates robust protein isolation schemes that simultaneously reduce non-specific protein contaminants while maintaining *bona fide* protein partners throughout purification.
• The TPL family of corepressors have widespread involvement in regulating plant gene expression and have been shown to interact with a diverse group of proteins in previous yeast-two-hybrid screens [2] and more direct studies (e.g. [3], [4]).
• Here is the first reported attempt at isolating TPL complexes from transgenic plants using formaldehyde to stabilize labile interactions and a TAP approach to reduce association of non-specific proteins.
• This reproducible dataset of proteins may be useful as a comparative list to remove false interactors from proteomic datasets.

## 1 Data

**Fig. 1:**
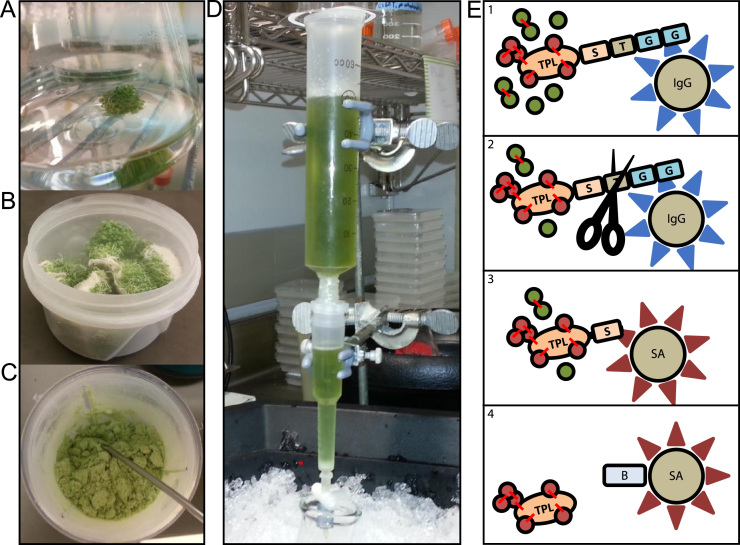
Plant material and pulldown procedure. (A) Culture flask after 12 days of growth, showing formation of Arabidopsis plant rafts floating on top of media. (B) One replicate of harvested TPL-GS-TAG plants. (C) Material after grinding in blender prior to addition of protein extraction buffer. (D) Column constructed for TAP. Red dot indicates IgG Sepharose® bed. Protein extract solution was added to the 50 mL syringe barrel and collected in a beaker on ice after flowing through the beads. (E) TAP procedure: (1) Whole protein extract is incubated with IgG Sepharose® which will bind the protein G portion of the tag. Red lines represent crosslinking by formaldehyde. Red filled circles indicate interacting proteins while, green circles indicate contaminating proteins. The GS-Tag is shown, with “G” indicating the protein G moiety, “T” is the TEV protease cleavable linker, “S” is the streptavidin binding peptide. (2) After washing the IgG-bound fraction, AcTEV protease cleaves the TEV linker. The eluted fraction contains TPL-GS-TAG. (3) The protease-treated elution is added to streptavidin beads. (4) After washing the bound sample, biotin is added to release TPL-GS-TAG from the streptavidin beads.

**Fig. 2:**
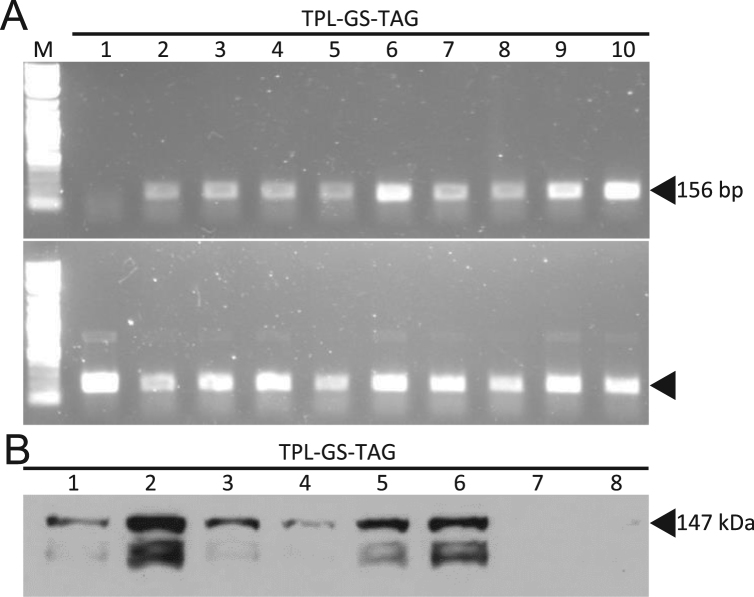
RNA and protein expression of TPL-GS-TAG in transgenic Arabidopsis. (A) QPCR showing ten T1 lines. The upper gel shows amplification of TPL-GS-TAG, and the lower gel shows the UBC21 control. Transgenic lines 6 and 10 were selected for the T2 generation. (B) Protein expression of TPL-GS-TA in the T2 generation. Twenty-five micrograms of total protein from each line were analyzed. Seeds from transgenic line 2 were used for generation of plant material for TAP.

**Fig. 3:**
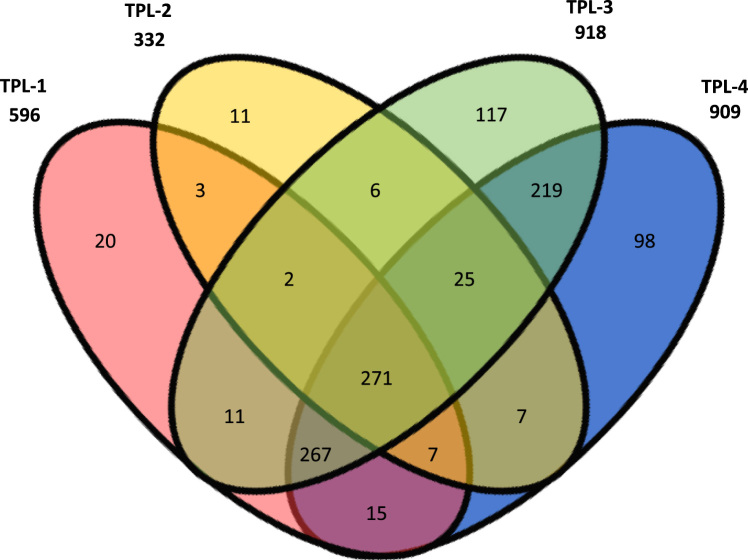
Proteins identified in each TPL-GS-TAG replicate. The Venn diagram indicates common proteins identified and the total number of proteins identified in each data set is indicated. For confident peptide identification, the FDR was set to 1% and protein identification required a minimum of two unique peptides per protein.

Transgenic *Arabidopsis* plants were generated and screened at the mRNA and protein level for the expression of TOPLESS-GS-TAG (Fig. 2). One high-expression TOPLESS-GS-TAG line was selected, and four independent TAP experiments were conducted to generate replicate datasets. Fig. 1 shows the plant material and workflow for protein extraction. The number of proteins identified in each replicate is shown in Fig. 3. Supplemental files 2–5 contain the filtered data sets for each TPL-GS-TAG replicate. Supplemental file 6 is a list of proteins identified in all four replicates, with scores and peptide information from each replicate listed in order. Supplemental file 7 contains proteins identified in three replicates. Supplemental file 8 shows the Gene Ontology (GO) pathway enrichment of the 271 proteins identified in all four replicates.

## 2 Experimental design, materials, and methods

### 2.1 Constructs utilized and *Agrobacterium*-mediated transformation of *Arabidopsis*

The GS-TAG was selected to purify TPL from *Arabidopsis* extracts as it allows two-step purification of the target protein [1]. The TPL sequence was amplified from *Arabidopsis* whole plant cDNA using the SuperScript™ III First-Strand Synthesis System (Invitrogen™). TPL was cloned into the Gateway® pENTR4™ vector (Invitrogen™) using the restriction sites NcoI and EcorV and the In-Fusion® kit (Clontech) according to the manufacturer׳s instructions. Sequence amplification and cloning into pENTR4™ were designed to ensure that after Gateway® cloning, TPL would be in-frame in the supplied destination vector pKCTAP [1]. Formation of the final TPL-GS-TAG vector was completed using the methodology outlined in [1] and the manufacturer׳s instructions for Gateway® cloning. Transformation of the *Agrobacterium* strain GV3101 was conducted via electroporation [5]. The growth of transformed *Agrobacterium*, *Arabidopsis* plants, and the floral dip method for transformation of *Arabidopsis* followed the standard procedure [6].

### 2.2 Selection of plants

T1 seeds were selected using 50 mg/L kanamycin according to the protocol outlined in [6]. Kanamycin resistant seedlings were transferred to soil, and a leaf sample was collected at four weeks for RNA extraction. Leaf samples were frozen in liquid nitrogen and pulverized using the 2000 Geno/Grinder® (SPEX® Sample Prep). RNA was extracted with the PureLink™ RNA Mini Kit (Ambion®) and quantified using the Epoch™ Microplate Spectrophotometer (BioTek®). Ten nanograms of total RNA was converted to cDNA using the SuperScript™ III One-Step RT-PCR System with Platinum™ *Taq* (Invitrogen™). An internal control (UBC21) and the target cDNA was amplified over 28 cycles, were separated via electrophoresis, and imaged using the Gel Logic 200 (Kodak). The two T1 plants with the highest RNA expression were selected by visually comparing the intensity of target to control bands (Fig. 2A). Primers utilized for “semi-quantitative” PCR are listed in Supplemental table 1.

One hundred milligrams of leaves were collected from eight kanamycin-resistant T2 seedlings to analyze TPL-GS-TAG protein expression. Tissue samples were frozen in liquid nitrogen and ground with the 2000 Geno/Grinder®, and suspended in 200 μL ice-cold grinding buffer (50 mM Tris-Cl pH 7.5, 150 mM NaCl, 0.1% NP-40, 0.1% SDS, 25 mM β-ME, 1x plant protease inhibitor cocktail (Sigma-Aldrich®)). Proteins were extracted on a rotating wheel at 4 °C for 5 min. Samples were spun at 14,000×*g* for 10 min and the supernatant collected. Total protein concentration was analyzed with the Bradford Protein Assay (Bio-Rad), and 25 μg of protein was run on a 10% SDS-gel. Proteins were transferred to Immuno-Blot™ PVDF membrane (Bio-Rad) using standard wet protein transfer methods. Protein expression was detected using the Peroxidase-Anti-Peroxidase Soluble Complex (PAP) (Sigma-Aldrich®) at a dilution of 1:5000. Proteins were detected using Immobilion™ Western Chemiluminescent HRP substrate (EMD Millipore) and exposure to X-ray film (Fig. 2B). The T2 plant with the highest protein expression was chosen for further protein isolation experiments.

### 2.3 Liquid-grown plant material and formaldehyde cross-linking

For the TAP experiment, four biological replicates of the selected T2 transgenic line were grown and purified using the following protocol. Plants were grown in 250 mL flasks containing 40 mL media (3.7 g/L MS salts, 10 g/L sucrose, 0.5 g/L MES, 50 μg/mL kanamycin, pH 5.7) for 16 days under a 16 h light/8 hr dark cycle with agitation on an orbital shaker set for 100 rpm (Fig. 1A). Fifteen milligrams of transgenic seeds that had been sterilized using 70% ethanol for 5 min, followed by 10% bleach for 15 min, and then rinsed three times with sterile Milli-Q® water were grown in each flask. Twelve flasks of plants were combined to produce a biological replicate sample.

Plants were harvested and rinsed with Milli-Q® water. Plants were then crosslinked by placing them in ice-cold PBS solution containing 1% formaldehyde and applying a vacuum for 10 min. The crosslinking reaction was quenched by transferring the plants to ice-cold PBS containing 125 mM glycine and placing them under vacuum for five minutes. Excess water was removed by transferring the plants to a 50 mL tube with holes punched in the bottom and spinning them at 1000 rpm for 30 s. Each sample produced approximately 40 g of plant material (Fig. 1B). Samples were stored at − 80 °C. after being frozen in liquid nitrogen.

### 2.4 Protein extraction

Protein extraction and purification followed the protocol outlined in [7] with modifications noted here. Samples were transferred to a nitrogen-chilled single-serve blender (Farberware®) and pulverized using the grinding blade for 30 s (Fig. 1C). An equal volume per weight of protein extraction buffer (25 mM Tris–HCl, pH 7.6, 15 mM MgCl2, 5 mM EGTA, 150 mM NaCl, 15 mM p-nitrophenylphosphate, 60 mM β-glycerophosphate, 0.1% (v/v) NP-40, 0.1 mM sodium vanadate, 1 mM NaF, 1 mM dithiothreitol (DTT), 1 mM PMSF, 1 μM E64, and plant protease inhibitor cocktail (Sigma-Aldrich®)) was added and mixed by stirring with a spatula for one minute. Samples were incubated on ice for 15 min and then divided into ice-cold 30 mL Oakridge tubes and spun at 39,000×*g* for 40 min at 4 °C. After centrifugation, the supernatant was poured through cheesecloth, followed by filtration through a 0.45 μm filter.

### 2.5 Tandem affinity purification

Unless otherwise noted, all solutions and protein handling steps were at 4 °C. An overview of the TAP procedure is shown in Fig. 1E. A column was constructed to maximize interaction between the antibody resin and the large volume (~ 80 mL) of protein extract by attaching a Poly-Prep® (Bio-Rad) column to a 50 mL sterile syringe barrel. IgG Sepharose® 6 fast-flow beads (GE Healthcare) were equilibrated as described in [7] on the homemade column. The filtered extract was then slowly passed over the beads four times over the course of an hour (Fig. 1D).

The 50 mL syringe was removed and the beads located in the Poly-Prep® column were washed as described [7]. The AcTEV protease treatment step was conducted in the Poly-Prep® column by plugging both ends. Collection of the elution fractions was done by placing the column in a 50 mL tube and centrifuging at 1000×*g* for 1.5 min at 4 °C. The TEV elution was incubated with pre-equilibrated High-Capacity Streptavidin Agarose Resin (Pierce™) (70 μL) and wash/elution steps were conducted as described [7].

### 2.6 Sample precipitation and SDS-PAGE

To precipitate the proteins, 117 μL of 100% TCA was added to each sample and mixed by inverting the tube five times. The tubes were kept on ice overnight, and the precipitated proteins were collected via centrifugation at 14,000×*g* for 40 min at 4 °C. The supernatant was removed, 500 μL of ice-cold 80% acetone was added, and the samples were stored overnight at − 20 °C. The following day, the samples were again spun at 14,000×*g* for 20 min at 4 °C. The acetone was removed, an additional 500 μL of ice-cold 80% acetone was added, and centrifugation step was repeated. The 80% acetone was removed, and the pellets were rinsed with 500 μL of ice-cold 100% acetone. Samples underwent a final 14,000×*g* centrifugation for 20 min at 4 °C, the acetone was removed, and residual acetone was evaporated by placing the tubes in a fume hood for 5 min.

Protein pellets were resuspended in 30 μL of SDS sample buffer without dye (50 mM Tris-Cl pH 6.8, 2% SDS, 10% glycerol, 100 mM DTT). The samples were heated at 99 °C for 10 min and then centrifuged at 14,000×*g* for 5 min. The samples were loaded onto a 10% Mini-PROTEAN® TGX™ stain-free gel (Bio-Rad) and run for 20 min. The gel was rinsed in MilliQ® water three times for 10 min and then stained in Bio-Safe™ Coomassie G-250 Stain (Bio-Rad) for 2 h with gentle agitation. Background Coomassie stain was removed by washing twice with Milli-Q® water for 10 min and then leaving it overnight in Milli-Q® water with gentle agitation on a shaker platform.

### 2.7 Removal of Coomassie stain and reduction/alkylation of gel pieces

Removal Coomassie stain, reduction and alkylation, and trypsin digestion followed the protocol in [8] with slight modifications noted here. Following tryptic digestion, gel fragments were treated twice with 50% acetonitrile and all three supernatants were combined to generate the peptide extraction solution. The extract was then lypholized in a CentriVap (Labconco). Samples were resuspended in 30 μL of 0.1% trifluoroacetic acid (TFA) with 10 min of sonication followed by 10 min of vortexing. The samples were then desalted via C-18 ZipTip® pipette tips (EMD Millipore). ZipTips® were equilibrated three times each with 10 μL of 100% acetonitrile, 10 μL of 50% acetonitrile/0.1% TFA, and 0.1% of TFA. The entire sample was passed through the ZipTip® three times. The ZipTip® was washed three times with 10 μL of 0.1% TFA, and three 10 μL volumes of 50% acetonitrile/0.1% TFA were used to elute the peptides. The samples were then dehydrated in the CentriVap for 15 min. The pellets were resuspended in 13 μL of 0.1% formic acid and frozen at − 80 °C until LC–MS.

### 2.8 Liquid chromatography tandem mass spectrometry

LC–MS was conducted with an EASY-nLC 1000 (Thermo Fisher Scientific™) coupled to an Orbitrap Elite ETD mass spectrometer (Thermo Fisher Scientific™). Peptide samples (10 μL) were loaded onto an Acclaim PepMap 100 pre-column (2 cm × 75 μm; 3 μm-C_18_) and separated with an EASY-spray analytical column (50 cm × 75 μm; 2 μm-C_18_) with the flow rate set to 350 nL/min. A gradient of 120 min using a binary pump system (solvent A: 0.1% formic acid in water; solvent B: 0.1% formic acid in 99.9% acetonitrile) was completed using 2–30% of B over 0–96 min; 30–100% of B over 96–108 min; and 100% B over 108–120 min.

Peptides were electrosprayed at 1800 V, and the capillary temperature was at 320 °C. The Nth Order Double Play mode was used for data acquisition. Full scan mass spectra were acquired over a range of 400–2000 Thompsons at a resolution of 120,000 (measured at *m*/*z* 400). The top 10 most intense ions were chosen for tandem mass spectrometry (MS/MS) with an isolation width of 2 *m*/*z* in CID mode and a resonance excitation normalized collision energy of 35. To avoid repeated analysis of identical peptides, dynamic exclusion was set to 10 s. For real-time internal calibration, lock mass of *m*/*z* 445.12003 (polysiloxane ion) was used.

### 2.9 Database searching and analysis

Proteome Discoverer™ version 1.4 (Thermo Fisher Scientific™) was used to process the raw files and spectra were searched using SEQUEST HT and the TAIR10_pep_20101214 *Arabidopsis* database [9]. The parameters were set as follows: allowance of a maximum of two missed tryptic cleavage sites, static modification of carbamidomethylation on cysteine residues (+ 57.021 Da), and dynamic modifications of oxidation of methionine (+ 15.996 Da) and deamination of asparagine (+ 0.984 Da). For identification of confident peptides, the false discovery rate (FDR, using a reverse database search) was set to 1%. Protein identification required at least two unique peptides per protein.

### 2.10 GO enrichment and pathway analysis

The agriGO software was used for GO enrichment and Singular Enrichment Analysis (SEA) [10], [11]. The input list contained all 271 common proteins identified by TAP of TPL. The background list was the *Arabidopsis* gene model TAIR9. We filtered the dataset to only contain enriched pathways with an FDR < 0.001% (Supplemental Fig. 8).
