# Supplementary material for: Proteomics dataset containing proteins that obscure identification of TOPLESS interactors in Arabidopsis
Source: Data Brief. 2018 Aug 29;20:909–16. doi: 10.1016/j.dib.2018.08.059 (PMC6138984; doi:10.1016/j.dib.2018.08.059)

We wish to confirm that there are no known conflicts of interest associated with this

publication and there has been no significant financial support for this work that could have

influenced its outcome.

We confirm that the manuscript has been read and approved by all named authors and that

there are no other persons who satisfied the criteria for authorship but are not listed. We

further confirm that the order of authors listed in the manuscript has been approved by all of

us.

We confirm that we have given due consideration to the protection of intellectual property

associated with this work and that there are no impediments to publication, including the

timing of publication, with respect to intellectual property. In so doing we confirm that we

have followed the regulations of our institutions concerning intellectual property.

We understand that the Corresponding Author is the sole contact for the Editorial process

(including Editorial Manager and direct communications with the office). He/she is

responsible for communicating with the other authors about progress, submissions of

revisions and final approval of proofs. We confirm that we have provided a current, correct

email address which is accessible by the Corresponding Author and which has been

configured to accept email from [schen@ufl.edu](mailto:schen@ufl.edu).


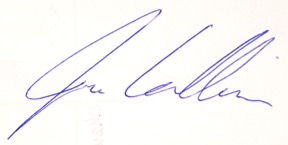


Signed by all authors as follows:

Joe Collins – 6/18/2018


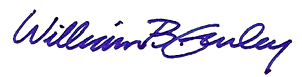


William B. Gurley – 6/18/2018


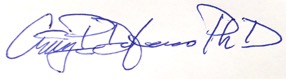


Craig Dufresne – 6/19/2018

Sixue Chen – Sixue Chen, 6/18/2018,
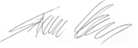

Supplement: Supplementary file 1 — Transparency document [file mmc1.docx]
