# Supplementary material for: Proteomics dataset containing proteins that obscure identification of TOPLESS interactors in Arabidopsis
Source: Data Brief. 2018 Aug 29;20:909–16. doi: 10.1016/j.dib.2018.08.059 (PMC6138984; doi:10.1016/j.dib.2018.08.059)
Supplement: Supplementary file 9 — Supplementary material [file mmc9.pdf]

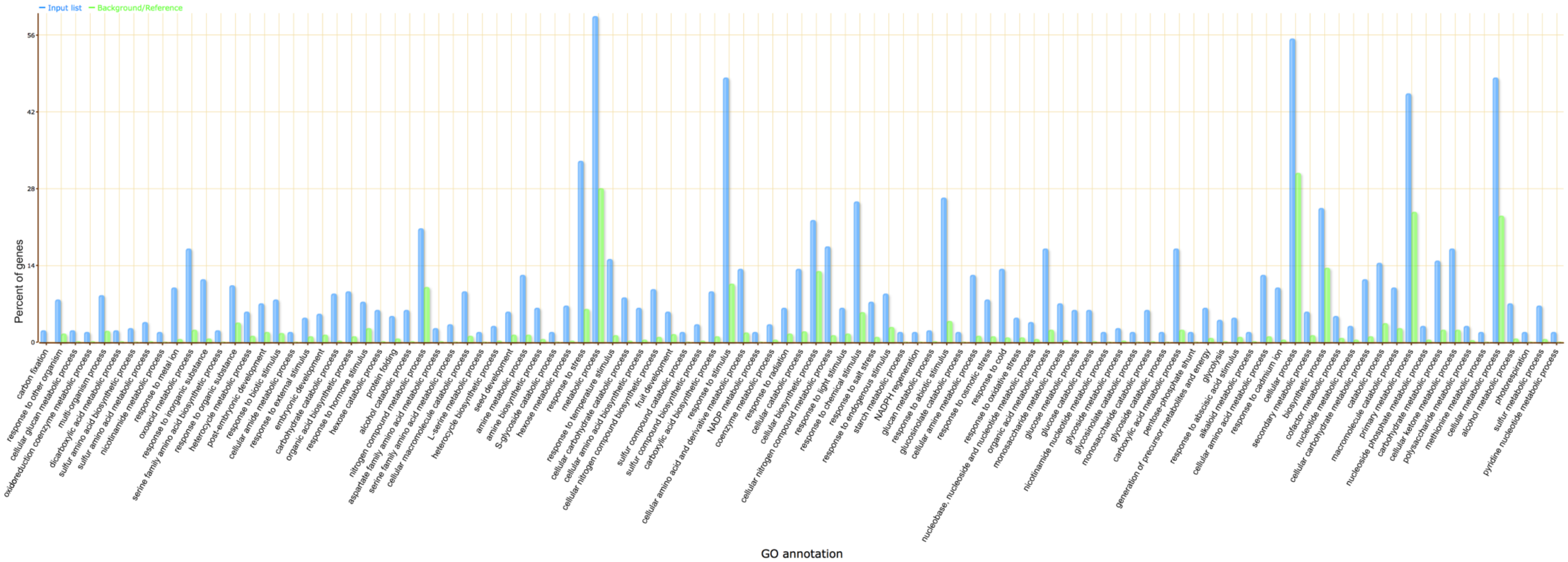

Supplemental Figure 8. AgriGO analysis of the 271 common proteins identified from TAP of TOPLESS [9, 10]. The percentage of genes identified in the study according to each pathway (blue) are compared to their corresponding percentage in the Arabidopsis genome (green).
